# Antibiotic Use in a Neonatal Intensive Care Unit Practicing Integrative Medicine—A Retrospective Analysis

**DOI:** 10.1089/jicm.2023.0001

**Published:** 2024-04-04

**Authors:** Jan Vagedes, Benedikt M. Huber, Mohammad Oli Al Islam, Katrin Vagedes, Matthias Kohl, Tido von Schoen-Angerer

**Affiliations:** ^1^ ARCIM Institute, Filderstadt, Germany.; ^2^ Department of Pediatrics, Filderklinik, Filderstadt, Germany.; ^3^ Department of Neonatology, University Hospital Tübingen, Tübingen, Germany.; ^4^ Center for Integrative Pediatrics, Fribourg Cantonal Hospital, Fribourg, Switzerland.; ^5^ Department of Community Health, Fribourg University, Fribourg, Switzerland.; ^6^ Institute of Precision Medicine, University Furtwangen, Furtwangen, Germany.

**Keywords:** neonatal intensive care, neonatal infection, neonatal sepsis, integrative medicine, antibiotic use rate, antibiotic stewardship, anthroposophic medicine

## Abstract

**Background:**

Antibiotic use in neonatal intensive care units (NICUs) remains high. Low antibiotic prescribing has been documented among physicians trained in complementary medicine. This study sought to identify if an NICU integrating complementary medicine has low antibiotic prescribing.

**Methods:**

We conducted a retrospective analysis at the level-2 NICU of the Filderklinik, an integrative medicine hospital in Southern Germany, to compare antibiotic use locally and internationally; to compare neonates with suspected infection, managed with and without antibiotics; and to describe use and safety of complementary medicinal products.

**Results:**

Among 7778 live births, 1086 neonates were hospitalized between 2014 and 2017. Two hundred forty-six were diagnosed with suspected or confirmed infection, their median gestational age was 40.3 weeks (range 29–42), 3.25% had a birthweight <2500 g, 176 were treated with antibiotics for a median duration of 4 days, 6 had culture-proven infection (0.77 per 1000 live births), and 2.26% of live births were started on antibiotics. A total of 866 antibiotic treatment days corresponded to 111 antibiotic days per 1000 live births and 8.8 antibiotic days per 100 hospital days. Neonates managed with antibiotics more often had fever and abnormal laboratory parameters than those managed without. Complementary medicinal products comprising 71 different natural substances were used, no side effect or adverse event were described. A subanalysis using the inclusion criteria of a recent analysis of 13 networks in Europe, North America, and Australia confirmed this cohort to be among the lowest prescribing networks.

**Conclusions:**

Antibiotic use was low in this NICU in both local and international comparison, while the disease burden was in the mid-range, confirming an association between integrative medicine practice and low antibiotic prescribing in newborns. Complementary medicinal products were widely used and well tolerated.

Clinical Trial Registration number: NCT04893343.

## Introduction

The use of antibiotics in neonatal intensive care units (NICUs) is under scrutiny not only because of rising antibiotic resistance in the neonatal population^1^ but also for the short- and long-term negative health effects of early antibiotic use,^2^ such as necrotizing enterocolitis,^3^ bronchopulmonary dysplasia,^4^ invasive candidiasis,^5^ nosocomial infection and mortality,^6^ and delayed initiation of breastfeeding of term newborns,^7^ as well as asthma and obesity later in life.^8–10^

The main challenge with NICU antibiotic prescribing is that the decision to start antibiotics largely depends on a clinician's interpretation of a clinical situation and laboratory parameters of limited significance in the context of a still unproven infection.^11,12^ Culture-positive sepsis is rare, with so-called culture-negative sepsis being reported to be 6–16 times higher.^13^ Culture-negative sepsis is a poorly defined and overdiagnosed condition, with numerous noninfectious conditions that mimic sepsis being far more common.^2^

Great variability has been observed in antibiotic prescribing NICUs with similar burden of proven infection, mortality, and other parameters^14^; the widest variation—31-fold in one study—has been observed among intermediate-level NICUs in the United States.^14^ The recent AENEAS study (Antibiotic Exposure for Suspected Neonatal Early-onset Sepsis) showed that the proportion of neonates started on antibiotics ranged from 1.18% to 12.45% among 13 hospital networks in Europe, North America, and Australia for the period 2014–2018.^15^ The AENAES study group proposed seven key indicators for reporting data on early-onset sepsis (EOS) and antibiotic use: (1) incidence of culture-proven EOS per 1000 live births, (2) EOS-associated mortality rate, (3) proportion of neonates started on antibiotics per 100 live births, (4) duration of antibiotic therapy, and (5) number of antibiotic days per 1000 live births, and description of the study by (6) gestational age and (7) all-cause mortality.

While NICU antibiotic use seems to decline, thanks to stewardship programs,^16,17^ more needs to be done. It is not known, if an integrative medicine approach, that is, the integration of complementary and conventional medicine, leads to lower NICU antibiotic prescribing. It has been shown that physicians trained in complementary medicine have lower antibiotic prescribing, including for children.^18–21^ This lower prescribing appears to be related to complementary medicine approaches to health, which focus on self-regulation and rebalancing within the organism, and the potential effectiveness of certain medications from complementary medicine for infectious and noninfectious diseases.^19^

Various complementary medicine approaches have been integrated and studied in NICU settings, including music therapy, massage therapy, osteopathy, acupuncture, and aromatherapy.^22–26^ In addition, two NICUs in Germany integrate a full range of both pharmacologic and nonpharmacologic complementary medicine interventions from anthroposophic medicine.^27^ One of them is at the Filderklinik, an anthroposophic, integrative medicine hospital in Southern Germany. The Filderklinik pediatric department has a long-standing practice of restrictive antibiotic use, which we previously confirmed for childhood community-acquired pneumonia.^21^

The aim of this retrospective study was to (1) identify antibiotic use and compare this to local and international data, (2) compare differences in morbidity and outcomes in neonates with infection, managed with and without antibiotics, and (3) describe the use of complementary medicinal products, as well as possible observed side effects.

## Methods

Study setting and population: A level-2 NICU in Filderstadt, in the South-Western state of Baden-Württemberg, Germany. In Germany, a level-2 perinatal center is defined as accepting neonates with an expected birth weight of >1250 g and >29 weeks gestational age and children of mothers with severe pregnancy-related disorders.^28^

Study procedure: We searched retrospectively all NICU admissions of age 0 to 1 month from January 1, 2014, to December 31, 2017. We further identified neonates who had an infectious disease diagnosis in their discharge letter. Infectious disease was defined as any ICD-10 diagnosis related to infections specific to the perinatal period (P35 congenital viral diseases; P36 bacterial sepsis of newborn; P37 other congenital infectious and parasitic diseases; P38 omphalitis of newborn; and P39 other infections specific to the perinatal period); congenital pneumonia (P23); and neonatal aspiration (P24).

We conducted a detailed retrospective analysis of hospital medical records for all neonates with infectious disease diagnosis, dividing them into neonates having received systemic antibiotic therapy and those who did not. In addition to clinical and laboratory parameters, we extracted use of complementary medicinal products (only products given internally) and reviewed the medical records for notes on side effects or adverse reactions for any medication.

Culture-proven infection was defined by positive blood and/or cerebrospinal fluid (CSF) culture. Contaminated cultures were defined by growth of bacteria usually considered contaminants with a decision to treat with antibiotics for less than 5 days; cultures with growth of coagulase-negative staphylococci and antibiotic therapy for more than 5 days were considered proven infection.^15^

The antibiotic exposure was calculated in several ways: (1) proportion of neonates started on antibiotics per 100 live births, (2) duration of antibiotic therapy, (3) number of antibiotic days per 1000 live births, and (4) antibiotic use rate for all NICU admissions as number of antibiotic treatment days per 100 hospital days.^29^

A subgroup analysis on antibiotic exposure was conducted to make our data set directly comparable to the AENEAS study.^15^ For this purpose, the antibiotic exposure was analyzed considering only infants born at a minimum gestational age of 34 weeks.

The federal state's quality assurance agency, Qualitätssicherung im Gesundheitswesen Baden-Württemberg GmbH, was contacted to obtain comparative data of the Filderklinik NICU with all level-2 NICUs in the same state of Baden-Württemberg in terms of patient demographics, morbidity, and antibiotic use.

Data analysis: The programming language R (R version 4.1.2)^30^ along with RStudio (Version 2021.09.1)^31^ was used to conduct all statistical analyses. The baseline values are expressed as descriptive statistics in absolute and relative frequency for categorical and binary variables. The median and interquartile range (IQR) is also included in the descriptive statistics for continuous variables. In addition to the descriptive statistics, comparison of continuous variables between antibiotic and no antibiotic was analyzed using Wilcoxon rank sum test and reported as median differences, 95% confidence interval (CI), *p*-value, and effect size “*r*.”^32^ Categorial variable comparison between antibiotic and no antibiotic was analyzed using the chi-square test and the *p*-value is reported.

The study was registered at clinicaltrials.gov with the identifier. The study was conducted according to the guidelines of the Declaration of Helsinki, was approved by the Ethics Committee of the University of Tübingen, Germany, and is reported here according to the STROBE-NI Statement.^33^ Patient consent was not applicable due to the retrospective and anonymized nature of the study.

## Results

**FIG. 1.**
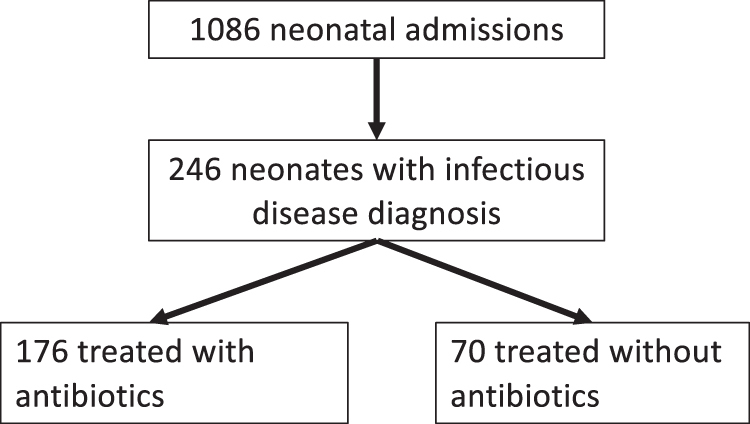
Flow diagram.

From 2014 to 2017, 7778 children were born at the Filderklinik and 1086 neonates were hospitalized in the NICU. Of these neonates, 246 had an ICD-10 infectious disease diagnosis (suspected or confirmed) at discharge. The majority, 176 (71.5%) neonates, were treated with intravenous antibiotics, and 70 (28.5%) were managed without antibiotics (Fig. 1 Flow Diagram).

Maternal, delivery, and birth parameters: Among the 246 neonates with infectious disease diagnosis, 36.6% were females, the median gestational age was 40.29 weeks (IQR 1.46, range 29–42), and 3.25% had a low birth weight (<2500 g). Other than a slightly higher gestational age in the antibiotic treatment group, there was no significant difference of maternal, delivery, and birth parameters between the two groups of neonates (Tables 1 and 2).

**Table 1.** Maternal and Delivery Parameters

		Total [*N* = 246]	Antibiotic [*n* = 176]	NAB [*n* = 70]	Test statistics*^a^*
Birth	Spontaneous	138 (56.10%)	99 (56.25%)	39 (55.71%)	*p* = 0.498
	Cesarean section	57 (23.17%)	38 (21.59%)	19 (27.14%)	
	Vacuum extraction/forceps delivery	48 (19.51%)	37 (21.02%)	11 (15.71%)	
Gestational diabetes	Yes	18 (7.32%)	14 (7.95%)	4 (5.71%)	*p* = 0.736
New-onset hypertension during pregnancy	Yes	4 (1.63%)	4 (2.27%)	0 (0.00%)	*p* = 0.476
Group B streptococcal screening	Positive	39 (15.85%)	33 (18.75%)	6 (8.57%)	*p* = 0.219
Rupture of membranes (hours before delivery)^b^	Median (IQR)	6.00 [16.00]	6.00 [16.00]	6.00 [11.50]	0.00 (−1.50; 2.00) *p* = 0.685; *r* = 0.03
Prolonged rupture of membranes	≥24 h	33 (13.41%)	22 (12.50%)	11 (15.71%)	
Maternal infection (any)	Present	2 (0.81%)	1 (0.57%)	1 (1.43%)	*p* = 1.000
Intrapartum antibiotics	Received	26 (10.57%)	17 (9.66%)	9 (12.86%)	*p* = 0.613
Amniotic fluid	Clear	144 (58.54%)	108 (61.36%)	36 (51.43%)	*p* = 0.473
	Light green/green	54 (21.95%)	37 (21.02%)	17 (24.29%)	
	Thick/green brown	24 (9.76%)	15 (8.52%)	9 (12.86%)	
	Bloody	2 (0.81%)	1 (0.57%)	1 (1.43%)	
Chorioamnionitis	Yes	7 (2.85%)	6 (3.41%)	1 (1.43%)	*p* = 0.676

^a^ Wilcoxon rank sum test performed and reported as median differences, 95% CI, *p*-value, and effect size “*r*” for “Continuous variables.” Otherwise, chi-square test performed and *p*-value reported.

^b^ Contentious variable; median and IQR is reported. Otherwise, absolute along with relative frequency reported.

CI, confidence interval; IQR, interquartile range; NAB, no antibiotic.

**Table 2.** Neonates at Birth

		Total [*N* = 246]	Antibiotic [*n* = 176]	NAB [*n* = 70]	Test statistics*^a^*
Twin pregnancy		8 (3.25%)	6 (3.41%)	2 (2.86%)	*p* = 1.000
Gender	Female	90 (36.59%)	65 (36.93%)	25 (35.71%)	*p* = 0.974
Gestational age (full weeks+days)^b^	Median (IQR)	40.29 [1.46]	40.57 [1.50]	40.00 [1.57]	−0.57 (−0.86; −0.14) *p* = 0.002; *r* = 0.20
Birthweight (gram)^b^	Median (IQR)	3560.00 [705.00]	3620.00 [687.50]	3490.00 [725.00]	−130.00 (−240.00; 65.00) *p* = 0.266; *r* = 0.07
Low birthweight	Birthweight ≥2500 g	236 (95.93%)	168 (95.45%)	68 (97.14%)	*p* = 0.479
	Low birthweight <2500 g	8 (3.25%)	7 (3.98%)	1 (1.43%)	
	Very low birthweight <1500 g	2 (0.81%)	1 (0.57%)	1 (1.43%)	
Apgar at 1 min	10	16 (6.50%)	12 (6.82%)	4 (5.71%)	*p* = 0.367
	9	121 (49.19%)	91 (51.70%)	30 (42.86%)	
	8	37 (15.04%)	23 (13.07%)	14 (20.00%)	
	4–7	52 (21.14%)	35 (19.89%)	17 (24.29%)	
	0–3	9 (3.66%)	8 (4.55%)	1 (1.43%)	
Apgar at 5 min	10	137 (55.69%)	100 (56.82%)	37 (52.86%)	*p* = 0.493
	9	54 (21.95%)	34 (19.32%)	20 (28.57%)	
	8	28 (11.38%)	21 (11.93%)	7 (10.00%)	
	4–7	20 (8.13%)	16 (9.09%)	4 (5.71%)	
	0–3	2 (0.81%)	1 (0.57%)	1 (1.43%)	
pH, umbilical artery^b^	Median (IQR)	7.21 [0.14]	7.20 [0.15]	7.21 [0.12]	0.01 (−0.01; 0.04) *p* = 0.314; *r* = 0.06
Base excess, umbilical artery^b^	Median (IQR)	−7.00 [5.95]	−7.10 [6.25]	−6.20 [5.00]	0.90 (−0.40; 2.10) *p* = 0.186; *r* = 0.08

^a^ Wilcoxon rank sum test performed and reported as median differences, 95% CI, *p*-value, and effect size “*r*.” Otherwise, Chi-square test performed and *p*-value reported.

^b^ Contentious variable; median and IQR is reported. Otherwise, absolute along with relative frequency reported.

Status of neonates at the time of admission and infectious disease parameters during hospitalization: The median age at admission was 0 days. Neonates in the antibiotic (AB) group were more likely to have fever, and had a slightly lower oxygen saturation and a higher C-reactive protein (CRP) value (Table 3). However, even in the no-antibiotic (NAB) group, CRP values of up to 38 mg/L at admission were recorded. The maximum neutrophil count over the time of hospitalization, but not the initial neutrophil count, was higher in the AB group.

**Table 3.** Status of Neonates at The Time of Admission

		Total [*N* = 246]	Antibiotic [*n* = 176]	NAB [*n* = 70]	Test statistics*^a^*
Age on admission (days)^b^	Median (IQR)	0.00 [1.00]	0.00 [1.00]	0.00 [1.00]	0.00 (−0.00;0.00) *p* = 0.131; *r* = 0.10
Temperature (C°)^b^	Median (IQR)	37.10 [0.90]	37.20 [0.98]	37.00 [0.70]	−0.20 (−0.40;0.00) *p* = 0.055; *r* = 0.12
Fever on admission (>38.0°C°)	Present	24 (9.76%)	22 (12.50%)	2 (2.86%)	*p* = 0.040
Heart rate (per minute)^b^	Median (IQR)	130.00 [25.50]	132.00 [25.25]	124.50 [22.75]	−7.50 (−12.00; −0.00) *p* = 0.042; *r* = 0.13
Respiratory rate (per minute)^b^	Median (IQR)	53.00 [21.00]	56.00 [22.00]	48.00 [18.50]	−8.00 (−10.00;0.00) *p* = 0.064; *r* = 0.12
0_2_ Saturation (%)^b^	Median (IQR)	97.00 [5.00]	97.00 [5.00]	98.00 [3.75]	1.00 (0.00;2.00) *p* = 0.001; *r* = 0.20
Abnormal skin color	Abnormal	61 (24.80%)	50 (28.41%)	11 (15.71%)	*p* = 0.113
Prolonged capillary refill time	>2 sec	95 (38.62%)	72 (40.91%)	23 (32.86%)	
Heart murmur	Absence documented	219 (89.02%)	157 (89.20%)	62 (88.57%)	*p* = 1.000
	Present	15 (6.10%)	11 (6.25%)	4 (5.71%)	
Lung auscultation	Normal lung auscultation	198 (80.49%)	142 (80.68%)	56 (80.00%)	*p* = 0.360
	Crackles	18 (7.32%)	13 (7.39%)	5 (7.14%)	
	Reduced breath sounds bilaterally	7 (2.85%)	6 (3.41%)	1 (1.43%)	
	Increased transmission of breath sounds	5 (2.03%)	2 (1.14%)	3 (4.29%)	
	Asymmetric breath sounds	3 (1.22%)	3 (1.70%)	0 (0.00%)	
Abdominal examination	Normal abdominal auscultation and palpation	211 (85.77%)	151 (85.79%)	60 (85.71%)	*p* = 0.916
	Distended	7 (2.85%)	5 (2.84%)	2 (2.86%)	
	Sparse bowel sounds	2 (0.81%)	1 (0.57%)	1 (1.43%)	
CRP (mg/L; on admission)^b^	Median (IQR)	7.0 [22.2]	8.7 [27.5]	4.0 [14.3]	−4.7 (−6.8; −0.00) *p* = 0.006; *r* = 0.17
CRP (mg/L; max. during hospitalization)^b^	Median (IQR)	21.8 [29.5]	28.8 [29.1]	10.5 [16.6]	−18.3 (−23.1; −13.8) *p* < 0.0001; *r* = 0.45
Neutrophil count (10^9^/L; on admission)^b^	Median (IQR)	65.70 [16.83]	66.10 [18.00]	64.70 [10.20]	−1.40 (−4.90;2.40) *p* = 0.496; *r* = 0.04
Neutrophil count (10^9^/L; max. during hospitalization)^b^	Median (IQR)	69.45 [15.00]	71.70 [14.80]	66.60 [13.60]	−5.10 (−8.60; −2.00) *p* = 0.002; *r* = 0.20
Leukocyte count (10^9^/L; on admission)^b^	Median (IQR)	16.10 [9.30]	15.85 [10.07]	17.50 [7.70]	1.65 (−1.40;2.40) *p* = 0.620; *r* = 0.03
Leukocyte count (10^9^/L; max. during hospitalization)^b^	Median (IQR)	18.40 [8.62]	19.20 [8.25]	17.90 [8.60]	−1.30 (−2.90;0.40) *p* = 0.157; *r* = 0.09
Blood culture	No growth	106 (43.09%)	88 (50.00%)	18 (25.71%)	*p* = 0.738
	Group B streptococci	2 (0.81%)	2 (1.14%)	0 (0.00%)	
	*Staphylococcus aureus* (MRSA type)	2 (0.81%)	2 (1.14%)	0 (0.00%)	
	*Staphylococcus epidermidis*	2 (0.81%)	1 (0.57%)	1 (1.43%)	
	*Staphylococcus hominis*	4 (1.63%)	3 (1.70%)	1 (1.43%)	
	*Staphylococcus auricularis*	1 (0.41%)	1 (0.57%)	0 (0.00%)	
	*Staphylococcus capitis*	1 (0.41%)	1 (0.57%)	0 (0.00%)	
Cerebrospinal fluid culture	No growth	3 (1.22%)	3 (1.70%)	0 (0.00%)	*p* = 0.083
	Positive	0 (0.00%)	0 (0.00%)	0 (0.00%)	NA
Urine culture	No growth	4 (1.63%)	4 (2.27%)	0 (0.00%)	NA
	Positive	1 (0.41%)	1 (0.57%)	0 (0.00%)	
Stool culture	No abnormal growth	6 (2.44%)	6 (3.41%)	0 (0.00%)	*p* = 0.014

^a^ Wilcoxon rank sum test performed and reported as median differences, 95% CI, *p*-value, and effect size “*r*.” Otherwise, chi-square test performed and *p*-value reported.

^b^ Contentious variable; median and interquartile range is reported. Otherwise, absolute along with relative frequency reported.

CRP, C-reactive protein.

A total of 121 blood cultures were drawn; among neonates exposed to antibiotics, 60% had a blood culture drawn. Positive blood cultures included group B streptococci (2), methicillin-resistant *Staphylococcus aureus* (2), and different coagulase-negative cocci (10); 8 of these were considered contamination and treated for less than 5 days and 2 treated for more than 5 days and thus considered proven infection. All CSF and urine cultures were negative. The number of culture-proven infection therefore was 6 (0.77 per 1000 live births).

Treatment of neonates: The proportion of neonates started on antibiotics per 100 live births was 2.26% (176 treated neonates for 7778 live births). There were 866 antibiotic treatment days for a total of 9817 hospital days (counting all NICU admissions), resulting in an antibiotic use rate of 8.8 antibiotic days per 100 hospital days. The number of antibiotic days per 1000 live births was 111.

The median antibiotic treatment duration was 4.0 days (IQR: 2.0) and the most common antibiotic treatment duration was 5–6 days (56.8%); 28.4% were treated for 2–4 days (Table 4). The most commonly used antibiotics were a combination of ampicillin and gentamicin (received by >96% of neonates in the AB group). No significant difference in the number of neonates receiving continuous positive airway pressure (CPAP) was observed between the AB and NAB group. Mechanical ventilation was used in 7 neonates in the AB and 1 neonate in the NAB group.

**Table 4.** Neonate Treatment

		Total [*N* = 246]	Antibiotic [*n* = 176]	NAB [*n* = 70]	Test statistics*^a^*
Antibiotic therapy duration	<48 h	4 (1.63%)	4 (2.27%)		NA
	2–4 days	50 (20.33%)	50 (28.41%)		
	5–6 days	100 (40.65%)	100 (56.82%)		
	≥ 7 days	18 (7.32%)	18 (10.23%)		
Ampicillin	Received	170 (69.11%)	170 (96.59%)		NA
Gentamicin	Received	169 (68.70%)	169 (96.02%)		NA
Cefotaxime	Received	5 (2.03%)	5 (2.84%)		NA
Flucoxacillin	Received	2 (0.81%)	2 (1.14%)		NA
Cefuroxime	Received	1 (0.41%)	1 (0.57%)		NA
Continous positive airway pressure CPAP	Received	52 (21.14%)	41 (23.30%)	11 (15.71%)	*p* = 0.222
Mechanical ventilation	Received	8 (3.25%)	7 (3.98%)	1 (1.43%)	*p* = 0.523
Hospitalization (days)^b^	Median (IQR)	6.00 [3.00]	6.00 [3.00]	3.00 [3.00]	−3.00 (−4.00; −2.00) *p* < 0.0001; *r* = 0.52

^a^ Wilcoxon rank sum test performed and reported as median differences, 95% CI, *p*-value, and effect size “*r*.” Otherwise, chi-square test performed and *p*-value reported.

^b^ Contentious variable; median and interquartile range is reported. Otherwise, absolute along with relative frequency reported.

Outcomes and discharge diagnoses: The hospitalization duration was twice as long in the AB group (6.0 days, IQR 3.0) (Table 4). No death occurred in either group. The five most common ICD-10 infectious disease diagnoses were bacterial sepsis of newborn (P36.9, 39.8%), infection specific to the neonatal period (P39.9, 25.2%), other bacterial sepsis of newborn (P36.8, 7.72%), congenital pneumonia (P23.9, 4.9%), and neonatal aspiration of meconium (P24.0, 4.5%) (see Supplementary Table S1 for full list of diagnoses).

Use of complementary medicinal products: Neonates in both the AB and the NAB group frequently received complementary medicinal products (Supplementary Table S2). Indications for prescription were generally not noted in medical records, but the range of products used indicates prescriptions for both infectious and noninfectious indications. Overall, 71 different natural substances were used in different preparations and dilutions. Route of administration included oral, inhalation, and intravenous (Supplementary Table S3). No side effect or adverse reaction was documented for neither conventional nor complementary medicinal products.

For the subanalysis to make the data comparable to the AENEAS study,^15^ we excluded 99 live births before 34 weeks of gestation and restricted analysis to neonates hospitalized within the first 7 days of life, thus presumably having started antibiotics in the first week of life. There were 7677 live births of minimum 34 weeks of gestation and 160 neonates treated with antibiotics, resulting in 2.08% of all live births in the Filderklinik exposed to antibiotics. With 2 positive cultures for group B streptococci, 1 methicillin-resistant *S. aureus*, and 8 coagulase-negative cocci treated for less than 5 days and 2 treated for more than 5 days, the number of culture-proven EOS was 5 and the incidence of culture-proven EOS per 1000 live births was 0.65. The number of antibiotic days was 772, resulting in 100 antibiotic days per 1000 live births.

According to reports received by the state's quality assurance agency, Qualitätssicherung im Gesundheitswesen Baden-Württemberg GmbH, 14.3% (CI: 12.3–6.5) of neonates hospitalized at the Filderklinik NICU were exposed to antibiotics between 2014 and 2017 compared to 30.9% (CI: 29.8–32.1) across all level-2 NICUs in the same German state of Baden-Württemberg (personal communication with Qualitätssicherung im Gesundheitswesen Baden-Württemberg GmbH). Unfortunately, it was not possible to obtain additional information about the other level-2 NICUs to assess if patient populations were comparable in terms of demographics and morbidity or to calculate the antibiotic use rate across these centers.

### Limitations

A number of limitations apply to this study due to its retrospective design. Infectious disease cases and antibiotic treatment courses were identified through infectious disease discharge diagnoses. Discharge diagnosis coding has been shown as reliable for common infections in adults,^34^ but there could have been a bias toward overdiagnosis because of the diagnosis-related group billing system. Such bias could have lowered the antibiotic use rate among the hospitalized neonates, but not the proportion of neonates started on antibiotics per live births. Possible overdiagnosis makes it unlikely that antibiotic treatment courses would have been missed in our analysis.

The more specific coding might also have been biased: The majority (55.1%) in the AB group was coded as neonatal sepsis (ICD-10 P36.9), while the majority in the NAB group (67.1%) was coded as infection specific to the neonatal period (ICD-10 P39.9). These coding decisions were probably influenced by the treatment, that is, infections were more likely coded as neonatal sepsis when antibiotics were used and coded as unspecified neonatal infection when antibiotics were not used. This bias had no influence on the group categorization and thus the outcomes of this study.

Other limitations linked to the retrospective design are possible missing data. For example, relevant clinical clues that guided clinicians to prescribe or withhold antibiotics may not have been documented. Not all neonates started on antibiotics had blood cultures drawn, resulting in a possible underdiagnosis of culture-proven sepsis.

The comparison with other level-2 NICUs in the same state was limited because patient demographics and morbidity could not be obtained for the other NICUs and data allowed only to compare the frequency of antibiotic prescription (prescription courses per 100 NICU admissions).

## Discussion

In this cohort of neonates from a hospital practicing an integrative medicine approach, the exposure to antibiotics was very low in international comparison. The antibiotic exposure in 13 networks in Europe, North America, and Australia, as reported by the AENEAS study, ranged from 1.18% to 12.45% of neonates per 1000 live births,^15^ while the Filderklinik exposure was 2.08% when using the same inclusion criteria. The disease burden of 0.65 EOS cases per 1000 live births was in the mid-range when compared to the range of 0.18–1.45 in the AENEAS study.

The antibiotic use rate (antibiotic days/hospital days) of 8.8% had no comparator in the AENEAS study, but was in the low range when compared to a cross-sectional survey in the United States, which found a highly variable antibiotic use rate ranging from 2.4% to 97.1% (median 24.5%).^29^ Antibiotic use of the Filderklinik NICU was also low in local comparison with 14.3% of hospitalized neonates receiving an antibiotic treatment course compared to an average 30.9% across all level-2 NICUs in the same state in Germany.

With a median age of 0 days at admission, the vast majority of neonates had suspected early-onset neonatal infections, including EOS. Our analysis found that 28.5% (70 out of 246) of neonates with a suspected infectious disease were managed without antibiotics. This does not mean that true neonatal infections can be managed without antibiotics; it simply shows that in a number of situations of suspected infection, careful monitoring without antibiotics is possible.

Vice versa, we do not know from this retrospective analysis if antibiotics were truly needed in all cases in the AB group—with 29 neonates treated per culture-positive sepsis and culture-negative sepsis being a poorly defined entity, there remains substantial overtreatment, as is true for all NICUs.^2^ Not surprisingly, neonates started on antibiotics were slightly sicker in terms of clinical appearance, laboratory parameters, and need for respiratory support than those who managed without. Nevertheless, the NAB group also included children with abnormal clinical and laboratory parameters and in need of respiratory assistance.

There were two lessons learnt for the Filderklinik NICU from this study. First, blood cultures need to be drawn in all neonates with suspected infection. Second, the most common antibiotic treatment duration was 5–6 days. The number of antibiotic days could be further reduced by implementing 48-h treatment courses for rule-out sepsis situations, and 5 days for culture-negative pneumonia and culture-negative sepsis.^35–37^

To our knowledge, this is the first report of antibiotic use in an NICU practicing integrative medicine. A large variety of complementary medicinal products from natural substances were used in both the AB and NAB group, in safe dilutions of 1:100 or higher in almost all cases. Neither the benefit of these complementary medicinal products nor their impact on antibiotic treatment decisions can be assessed from this study.

While benefit is possible, it should be noted that some networks such as in Stockholm, Sweden,^15^ had lower antibiotic use than this network and without resorting to complementary medicine. The lack of documented side effects or adverse reactions is reassuring. Regardless of any proven effect of complementary medicinal products, physicians trained in complementary medicine are known to be more restrictive in antibiotic prescribing, including hospitalized children,^21^ putting more emphasis on reinforcing natural healing capacity.^19^ Therefore, the hypothesis that complementary therapies—in addition to other antimicrobial stewardship programs—can contribute to decrease the burden of antibiotic treatment in neonates should be further explored.

## Conclusions

Antibiotic use was low in this integrative medicine NICU in both local and international comparison, confirming an association, but no causality between integrative medicine practice and low antibiotic prescription in newborns. Complementary medicinal products were widely used for infectious and noninfectious indications and well tolerated. Future prospective studies should test whether or not the lower exposure to antibiotics presented in this study can be attributed to the additional use of complementary medicinal products.

## Supplementary Material

Supplemental data

Supplemental data

Supplemental data

## Data Availability

Raw data are available through the OSF Registry at https://osf.io/b8etw/
