# Supplementary material for: Antibiotic Use in a Neonatal Intensive Care Unit Practicing Integrative Medicine—A Retrospective Analysis
Source: J Integr Complement Med. 2024 Apr 4;30(4):394–402. doi: 10.1089/jicm.2023.0001 (PMC11001955; doi:10.1089/jicm.2023.0001)
Supplement: Supplemental data [file Suppl_TableS1.docx]

Supplementary Table S1: ICD-10 Diagnoses at Discharge

| **ICD-10 Diagnosis** | ***Total***  ***[N=246]*** | ***Antibiotic***  ***[n=176]*** | ***No-Antibiotic***  ***[n=70]*** |
| --- | --- | --- | --- |
| Bacterial sepsis of newborn, unspecified P36.9 | 98(39.84%) | 97(55.11%) | 1(1.43%) |
| Infection specific to the perinatal period, unspecified P39.9 | 62(25.20%) | 15(8.52%) | 47(67.14%) |
| Other bacterial sepsis of newborn P36.8 | 19(7.72%) | 18(10.23%) | 1(1.43%) |
| Congenital pneumonia, unspecified P23.9 | 12(4.88%) | 8(4.55%) | 4(5.71%) |
| Neonatal aspiration of meconium P24.0 | 11(4.47%) | 7(3.98%) | 4(5.71%) |
| Other specified infections specific to the perinatal period  P39.8 | 8(3.25%) | 5(2.84%) | 3(4.29%) |
| Sepsis of newborn due to streptococcus, group B P36.0 | 4(1.63%) | 4(2.27%) | 0(0.00%) |
| Other respiratory distress of newborn P22.8 | 3(1.22%) | 1(0.57%) | 2(2.86%) |
| Neonatal skin infection P39.4 | 3(1.22%) | 3(1.70%) | 0(0.00%) |
| Respiratory distress syndrome of newborn P22.0 | 2(0.81%) | 1(0.57%) | 1(1.43%) |
| Congenital pneumonia due to other organisms P23.8 | 2(0.81%) | 2(1.14%) | 0(0.00%) |
| Bacterial sepsis of newborn P36 | 2(0.81%) | 2(1.14%) | 0(0.00%) |
| Sepsis of newborn due to Staphylococcus aureus P36.2 | 2(0.81%) | 2(1.14%) | 0(0.00%) |
| Omphalitis of newborn with or without mild haemorrhage P38 | 2(0.81%) | 1(0.57%) | 1(1.43%) |
| Fetus and newborn affected by chorioamnionitis P02.7 | 1(0.41%) | 1(0.57%) | 0(0.00%) |
| Intrauterine hypoxia first noted during labour and delivery P20.1 | 1(0.41%) | 1(0.57%) | 0(0.00%) |
| Intrauterine hypoxia, unspecified P20.9 | 1(0.41%) | 1(0.57%) | 0(0.00%) |
| Congenital pneumonia due to other bacterial agents P23.6 | 1(0.41%) | 1(0.57%) | 0(0.00%) |
| Neonatal aspiration of amniotic fluid and mucus P24.1 | 1(0.41%) | 0(0.00%) | 1(1.43%) |
| Pneumothorax originating in the perinatal period P25.1 | 1(0.41%) | 1(0.57%) | 0(0.00%) |
| Cyanotic attacks of newborn P28.2 | 1(0.41%) | 0(0.00%) | 1(1.43%) |
| Respiratory condition of newborn, unspecified P28.9 | 1(0.41%) | 1(0.57%) | 0(0.00%) |
| Sepsis of newborn due to other and unspecified staphylococci P36.3 | 1(0.41%) | 1(0.57%) | 0(0.00%) |
| Other infections specific to the perinatal period P39 | 1(0.41%) | 1(0.57%) | 0(0.00%) |
| Neonatal conjunctivitis and dacryocystitis P39.1 | 1(0.41%) | 0(0.00%) | 1(1.43%) |
| Syndrome of infant of a diabetic mother P70.1 | 1(0.41%) | 1(0.57%) | 0(0.00%) |
| Other neonatal hypoglycaemia P70.4 | 1(0.41%) | 0(0.00%) | 1(1.43%) |
| Slow feeding of newborn P92.2 | 1(0.41%) | 0(0.00%) | 1(1.43%) |
| Other specified conditions originating in the perinatal period P96.8 | 1(0.41%) | 0(0.00%) | 1(1.43%) |
