# Supplementary material for: Antibiotic Use in a Neonatal Intensive Care Unit Practicing Integrative Medicine—A Retrospective Analysis
Source: J Integr Complement Med. 2024 Apr 4;30(4):394–402. doi: 10.1089/jicm.2023.0001 (PMC11001955; doi:10.1089/jicm.2023.0001)
Supplement: Supplemental data [file Suppl_TableS2.docx]

Supplementary Table S2: Complementary Medicinal Products

| ***Medicinal Product*** | ***Total***  ***[N=246]*** | ***Antibiotic***  ***[n=176]*** | ***No-Antibiotic***  ***[n=70]*** |
| --- | --- | --- | --- |
| *Achillea D3* | 9 (3.66%) | 6 (3.41%) | 3 (4.29%) |
| *Aconitum comp. (Aconitum D29, Belladonna D29, Toxicodendron D29)* | 2 (0.81%) | 1 (0.57%) | 1 (1.43%) |
| *Aconitum napellus D30* | 56 (22.76%) | 42 (23.86%) | 14 (20.00%) |
| *Aconitum napellus D6* | 1 (0.41%) | 1 (0.57%) | 0 (0.00%) |
| *Adonis comp. (Adonis vernalis D2, Crataegus D2)* | 1 (0.41%) | 1 (0.57%) | 0 (0.00%) |
| *Amnion GI D30* | 9 (3.66%) | 6 (3.41%) | 3 (4.29%) |
| *Amnion Gl D12* | 1 (0.41%) | 1 (0.57%) | 0 (0.00%) |
| *Anaemodoron® RhD2 (Fragaria vesca D2, Urtica doica D2)* | 4 (1.63%) | 4 (2.27%) | 0 (0.00%) |
| *Apis Belladonna (Apis D4, Belladonna D3)* | 2 (0.81%) | 2 (1.14%) | 0 (0.00%) |
| *Argentum D29 /Echinacea D1* | 3 (1.22%) | 3 (1.70%) | 0 (0.00%) |
| *Argentum D30 /Echinacea D6* | 121 (49.19%) | 98 (55.68%) | 23 (32.86%) |
| *Argentum Echinacea (potency not specified)* | 82 (33.33%) | 54 (30.68%) | 28 (40.00%) |
| *Argentum metallicum praeparatum D20* | 2 (0.81%) | 1 (0.57%) | 1 (1.43%) |
| *Arnica D12* | 2 (0.81%) | 2 (1.14%) | 0 (0.00%) |
| *Arnica D6* | 27 (10.98%) | 20 (11.36%) | 7 (10.00%) |
| *Arnica Rh D20* | 4 (1.63%) | 2 (1.14%) | 2 (2.86%) |
| *Arnica D30* | 12 (4.88%) | 7 (3.98%) | 5 (7.14%) |
| *Arnica D20/ArgentumD20/Stibium arsenicosum D6* | 17 (6.91%) | 15 (8.52%) | 2 (2.86%) |
| *Aurum comp. (Aurum D6, Myrrha D3, Olibanum D3)* | 1 (0.41%) | 1 (0.57%) | 0 (0.00%) |
| *Avena sativa D6* | 1 (0.41%) | 1 (0.57%) | 0 (0.00%) |
| *Belladonna/Chamomilla (Belladonna D5, Chamomilla D2)* | 1 (0.41%) | 1 (0.57%) | 0 (0.00%) |
| *Belladonna D6* | 1 (0.41%) | 1 (0.57%) | 0 (0.00%) |
| *Bryonia (potency not specified)* | 1 (0.41%) | 1 (0.57%) | 0 (0.00%) |
| *Bryonia/Aconitum (Bryonia D7, Aconitum D5)* | 7 (2.85%) | 6 (3.41%) | 1 (1.43%) |
| *Bryophyllum 5%* | 11 (4.47%) | 10 (5.68%) | 1 (1.43%) |
| *Bryophyllum 50%* | 3 (1.22%) | 2 (1.14%) | 1 (1.43%) |
| *Bryophyllum Argento Cultum Rh D3* | 5 (2,03%) | 4 (2,27%) | 1 (1.43%) |
| *Bryophyllum D5 / Conchae D7* | 4 (1.63%) | 4 (2.27%) | 0 (0.00%) |
| *Calendula D4* | 3 (1.22%) | 3 (1.70%) | 0 (0.00%) |
| *Carbo Betulae D6* | 55 (22.36%) | 55 (31.25%) | 0 (0.00%) |
| *Carbo betulae D10* | 1 (0.41%) | 1 (0.57%) | 0 (0.00%) |
| *Carbo betulae D12* | 4 (1.63%) | 4 (2.27%) | 0 (0.00%) |
| *Carbo Betulae D20* | 2 (0.81%) | 2 (1.14%) | 0 (0.00%) |
| *Cardiodoron® (Onopordum, Hyoscyamus niger, Primula veris)* | 28 (11.38%) | 23 (13.07%) | 5 (7.14%) |
| *Cerebrum comp A (Cerebellum D8, Corpora quadrigemina D8, Hypophysis D8, Iris D8, Medulla oblangat D8, Nervus opticus D8, Retina et Chorioidea D8, Thalmaus D8)* | 4 (1.63%) | 3 (1.70%) | 1 (1.43%) |
| *Cerebrum Comp B (Aurum D6, Cerebellum D7, Corpora quadrigemina D7, Hypophysis D7, Iris D7, Medulla oblangat D7, Myrrha D3, Nervus opticus D7, Olibanum D3, Retina et Chorioidea D7, Thalmaus D7)* | 1 (0.41%) | 0 (0.00%) | 1 (1.43%) |
| *Chamoilla Cupro culta Rh D3* | 37 (15,04%) | 35 (19,89%) | 2 (2,86%) |
| *Chamomilla D6* | 1 (0.41%) | 1 (0.57%) | 0 (0.00%) |
| *Chelidonium Ferro cultum Rh D3* | 2 (0.81%) | 2 (1.14%) | 0 (0.00%) |
| *Cuprum aceticum comp. (Cuprum aceticum D5, Nicotiana tabacum D9, Renes D5)* | 1 (0.41%) | 1 (0.57%) | 0 (0.00%) |
| *Equisetum (potency not specified)* | 2 (0.81%) | 1 (0.57%) | 1 (1.43%) |
| *Equisetum arvense Rh D6* | 19 (7.72%) | 15 (8.52%) | 4 (5.71%) |
| *Equisetum arvense Rh D3* | 3 (1,22%) | 3 (1.70%) | 0 (0.00%) |
| *Gentiana Magen Globuli (Artemisia absinthium ø, Gentiana lutea ø, Strychnos nux-vomica D4, Taraxacum ø)* | 11 (4.47%) | 9 (5.11%) | 2 (2.86%) |
| *Geum urbanum Rh D3* | 1 (0.41%) | 1 (0.57%) | 0 (0.00%) |
| *Hepar/Stannum I (Hepar D5, Stannum D9)* | 1 (0.41%) | 1 (0.57%) | 0 (0.00%) |
| *Hypericum perforatum D12* | 1 (0.41%) | 0 (0.00%) | 1 (1.43%) |
| *Hypericum perforatum D30* | 1 (0.41%) | 1 (0.57%) | 0 (0.00%) |
| *Ignatia D30* | 1 (0.41%) | 1 (0.57%) | 0 (0.00%) |
| *Melissa Cupro culta Rh D3* | 1 (0.41%) | 1 (0.57%) | 0 (0.00%) |
| *Meteoreisen Globuli velati (Ferrum sidereum D11, Phosphor D5, Quarz D11)* | 5 (2.03%) | 4 (2.27%) | 1 (1.43%) |
| *Nicotiana comp. (Carbo vegetabilis D19, Chamomilla D2, Nicotiana D9)* | 1 (0.41%) | 1 (0.57%) | 0 (0.00%) |
| *Nux vomica D4* | 10 (4.07%) | 6 (3.41%) | 4 (5.71%) |
| *Okoubaka D6* | 1 (0.41%) | 1 (0.57%) | 0 (0.00%) |
| *Olibanum comp. (Aurum D30, Myrrha D6, Olibanum D12)* | 17 (6.91%) | 12 (6.82%) | 5 (7.14%) |
| *Opium D30* | 3 (1.22%) | 3 (1.70%) | 0 (0.00%) |
| *Passiflora comp. (Crataegus D2, Passiflora D2, Salix alba D2)* | 7 (2.85%) | 6 (3.41%) | 1 (1.43%) |
| *Phosphor D8* | 12 (4.88%) | 11 (6.25%) | 1 (1.43%) |
| *Phosphor D30* | 4 (1.63%) | 2 (1.14%) | 2 (2.86%) |
| *Phosphorus D6 / Tartarus Stibiatus D4* | 2 (0.81%) | 1 (0.57%) | 1 (1.43%) |
| *Plexus Brachialis Bovis D6* | 1 (0.41%) | 1 (0.57%) | 0 (0.00%) |
| *Pneumodoron I (Aconitum D2, Bryonia D2)* | 1 (0.41%) | 1 (0.57%) | 0 (0.00%) |
| *Pneumodoron Ii (Phosphor D4, Tartarus D4)* | 1 (0.41%) | 1 (0.57%) | 0 (0.00%) |
| *Prunuseisen D3 (Prunus spinosa cum Ferro D3)* | 3 (1.22%) | 3 (1.70%) | 0 (0.00%) |
| *Pulmo/Vivianit comp. (Bryonia D5, Pulmo D16, Tartarus stibiatus D7, Vivianit D7)* | 47 (19.11%) | 40 (22.73%) | 7 (10.00%) |
| *Pulvis stomachicus cum Belladonna (Antimonit D3, Belladonna D3, Bismutum D3, Chamomilla D3)* | 3 (1.22%) | 2 (1.14%) | 1 (1.43%) |
| *Sambuccus comp. (Sambucus nigra e medulla D3, Sambucus nigra ex umbella D2, Terebinthina D4)* | 1 (0.41%) | 0 (0.00%) | 1 (1.43%) |
| *Stibium metallicum praeparatum D6* | 4 (1.63%) | 3 (1.70%) | 1 (1.43%) |
| *Tartarus stibiatus (potency not specified9* | 59 (23.98%) | 48 (27.27%) | 11 (15.71%) |
| *Urtica comp. (Conchae D6, Stannum D9, Urtica urens D2)* | 1 (0.41%) | 1 (0.57%) | 0 (0.00%) |
