# Supplementary material for: Antibiotic Use in a Neonatal Intensive Care Unit Practicing Integrative Medicine—A Retrospective Analysis
Source: J Integr Complement Med. 2024 Apr 4;30(4):394–402. doi: 10.1089/jicm.2023.0001 (PMC11001955; doi:10.1089/jicm.2023.0001)
Supplement: Supplemental data [file Suppl_TableS3.docx]

Supplementary Table S3: Complementary Medicine Products and Route of Administration

| ***Medicinal Product*** | ***Route of Administration*** | ***Total [N=246]*** |
| --- | --- | --- |
| *Achillea D3* | *PO* | 9 (3.66%) |
| *Aconitum comp. (Aconitum D29, Belladonna D29, Toxicodendron D29)* | *IV* | 2 (0.81%) |
| *Aconitum napellus D30* | *PO* | 27 (10.98%) |
|  | *IV* | 22 (8.94%) |
|  | *IV + PO* | 5 (2.03%) |
|  | *Not Mentioned* | 2 (0.81%) |
| *Aconitum napellus D6* | *Not Mentioned* | 1 (0.41%) |
| *Adonis comp. (Adonis vernalis D2, Crataegus D2)* | *PO* | 1 (0.41%) |
| *Amnion GI D30* | *PO* | 9 (3.66%) |
| *Amnion Gl D12* | *PO* | 1 (0.41%) |
| *Anaemodoron® Rh D2 (Fragaria vesca D2, Urtica doica D2)* | *PO* | 4 (1.63%) |
| *Apis Belladonna (Apis D4, Belladonna D3)* | *PO* | 1 (0.41%) |
|  | *Not Mentioned* | 1 (0.41%) |
| *Argentum D29 /Echinacea D1* | *PO* | 1 (0.41%) |
|  | *IV + PO* | 2 (0.81%) |
| *Argentum D30 /Echinacea D6* | *PO* | 35 (14.23%) |
|  | *IV* | 40 (16.26%) |
|  | *IV + PO* | 46 (18.70%) |
|  | *Not Mentioned* | 1 (0.41%) |
| *Argentum Echinacea (potency not specified)* | *PO* | 23 (9.35%) |
|  | *IV* | 29 (11.79%) |
|  | *IV + PO* | 27 (10.98%) |
|  | *Not Mentioned* | 3 (1.22%) |
| *Argentum metallicum praeparatum D20* | *PO* | 1 (0.41%) |
|  | *IV* | 1 (0.41%) |
| *Arnica D12* | *PO* | 2 (0.81%) |
| *Arnica D6* | *PO* | 25 (10.16%) |
|  | *IV* | 2 (0.81%) |
| *Arnica Rh D20* | *PO* | 1 (0.41%) |
|  | *IV* | 3 (1.22%) |
| *Arnica D30* | *PO* | 10 (4.07%) |
|  | *IV* | 1 (0.41%) |
|  | *IV + PO* | 1 (0.41%) |
| *Arnica D20/ArgentumD20/Stibium arsenicosum D6* | *IV* | 16 (6.50%) |
|  | *Not Mentioned* | 1 (0.41%) |
| *Aurum comp. (Aurum D6, Myrrha D3, Olibanum D3)* | *PO* | 1 (0.41%) |
| *Avena sativa D6* | *PO* | 1 (0.41%) |
| *Belladonna/Chamomilla (Belladonna D5, Chamomilla D2)* | *PO* | 1 (0.41%) |
| *Belladonna D6* | *PO* | 1 (0.41%) |
| *Bryonia (potency not specified)* | *PO* | 1 (0.41%) |
| *Bryonia/Aconitum (Bryonia D7, Aconitum D5)* | *PO* | 4 (1.63%) |
|  | *IV* | 2 (0.81%) |
|  | *IV + PO* | 1 (0.41%) |
| *Bryophyllum 5%* | *PO* | 3 (1.22%) |
|  | *IV* | 8 (3.25%) |
| *Bryophyllum 50%* | *PO* | 3 (1.22%) |
| *Bryophyllum Argento Cultum Rh D3* | *PO* | 3 (1.22%) |
|  | *IV* | 2 (0.81%) |
| *Bryophyllum D5 / Conchae D7* | *PO* | 1 (0.41%) |
|  | *IV* | 3 (1.22%) |
| *Calendula D4* | *PO* | 2 (0.81%) |
|  | *Not Mentioned* | 1 (0.41%) |
| *Carbo betulae D6* | *PO* | 50 (20.33%) |
|  | *IV + PO* | 1 (0.41%) |
|  | *Not Mentioned* | 4 (1.63%) |
| *Carbo betulae D6* | *Not Mentioned* | 1 (0.41%) |
| *Carbo betulae D10* | *Not Mentioned* | 1 (0.41%) |
| *Carbo betulae D12* | *PO* | 4 (1.63%) |
| *Carbo betulae D20* | *PO* | 1 (0.41%) |
|  | *IV* | 1 (0.41%) |
| *Cardiodoron® (Onopordum, Hyoscyamus niger, Primula veris)* | *PO* | 16 (6.50%) |
|  | *IV* | 3 (1.22%) |
|  | *IV + PO* | 8 (3.25%) |
|  | *Not Mentioned* | 1 (0.41%) |
| *Cerebrum comp A (Cerebellum D8, Corpora quadrigemina D8, Hypophysis D8, Iris D8, Medulla oblangat D8, Nervus opticus D8, Retina et Chorioidea D8, Thalmaus D8)* | *PO* | 3 (1.22%) |
|  | *IV* | 1 (0.41%) |
| *Cerebrum Comp B (Aurum D6, Cerebellum D7, Corpora quadrigemina D7, Hypophysis D7, Iris D7, Medulla oblangat D7, Myrrha D3, Nervus opticus D7, Olibanum D3, Retina et Chorioidea D7, Thalmaus D7)* | *PO* | 1 (0.41%) |
| *Chamoilla Rh D3* | *PO* | 8 (3.25%) |
|  | *Not Mentioned* | 2 (0.81%) |
| *Chamomilla Cupro culta Rh D3* | *PO* | 23 (9.35%) |
|  | *IV* | 1 (0.41%) |
|  | *Not Mentioned* | 3 (1.22%) |
| *Chamomilla D6* | *PO* | 1 (0.41%) |
| *Chelidonium Ferro cultum Rh D3* | *PO* | 2 (0.81%) |
| *Cuprum aceticum comp. (Cuprum aceticum D5, Nicotiana tabacum D9, Renes D5)* | *Inhalation* | 1 (0.41%) |
| *Equisetum (potency not specified)* | *IV* | 2 (0.81%) |
| *Equisetum arvense Rh D3* | *IV* | 3 (1.22%) |
| *Equisetum arvense Rh D6* | *PO* | 2 (0.81%) |
|  | *IV* | 12 (4.87%) |
|  | *IV + PO* | 4 (1.63%) |
|  | *Not Mentioned* | 1 (0.41%) |
| *Gentiana Magen Globuli (Artemisia absinthium ø, Gentiana lutea ø, Strychnos nux-vomica D4, Taraxacum ø)* | *PO* | 11 (4.47%) |
| *Geum urbanum Rh D3* | *PO* | 1 (0.41%) |
| *Hepar/Stannum I (Hepar D5, Stannum D9)* | *PO* | 1 (0.41%) |
| *Hypericum perforatum D12* | *Not Mentioned* | 1 (0.41%) |
| *Hypericum perforatum D30* | *PO* | 1 (0.41%) |
| *Ignatia D30* | *PO* | 1 (0.41%) |
| *Melissa Cupro culta Rh D3* | *PO* | 1 (0.41%) |
| *Meteoreisen Globuli velati (Ferrum sidereum D11, Phosphor D5, Quarz D11)* | *PO* | 5 (2.03%) |
| *Nicotiana comp. (Carbo vegetabilis D19, Chamomilla D2, Nicotiana D9)* | *PO* | 1 (0.41%) |
| *Nux vomica D4* | *PO* | 10 (4.07%) |
| *Okoubaka D6* | *PO* | 1 (0.41%) |
| *Olibanum comp. (Aurum D30, Myrrha D6, Olibanum D12)* | *PO* | 13 (5.28%) |
|  | *IV* | 4 (1.63%) |
| *Opium D30* | *PO* | 2 (0.81%) |
|  | *Not Mentioned* | 1 (0.41%) |
| *Passiflora comp. (Crataegus D2, Passiflora D2, Salix alba D2)* | *PO* | 6 (2.44%) |
|  | *Not Mentioned* | 1 (0.41%) |
| *Phosphor D30* | *PO* | 3 (1.22%) |
|  | *Not Mentioned* | 1 (0.41%) |
| *Phosphor D8* | *PO* | 10 (4.07%) |
|  | *Inhalation* | 1 (0.41%) |
|  | *IV* | 1 (0.41%) |
| *Phosphorus D6 / Tartarus stibiatus D4* | *PO* | 2 (0.81%) |
| *Plexus brachialis GI D6* | *PO* | 1 (0.41%) |
| *Pneumodoron I (Aconitum D2, Bryonia D2)* | *PO* | 1 (0.41%) |
| *Pneumodoron II (Phosphor D4, Tartarus D4)* | *PO* | 1 (0.41%) |
| *Prunuseisen D3 (Prunus spinosa cum Ferro D3)* | *PO* | 3 (1.22%) |
| *Pulmo/Vivianit comp. (Bryonia D5, Pulmo D16, Tartarus stibiatus D7, Vivianit D7)* | *PO* | 8 (3.25%) |
|  | *Inhalation* | 27 (10.98%) |
|  | *IV + Inhalation* | 5 (2.03%) |
|  | *IV* | 2 (0.81%) |
|  | *IV + PO* | 2 (0.81%) |
|  | *PO + Inhalation* | 2 (0.81%) |
|  | *IV + PO + Inhalation* | 1 (0.41%) |
| *Pulvis stomachicus cum Belladonna (Antimonit D3, Belladonna D3, Bismutum D3, Chamomilla D3)* | *PO* | 3 (1.22%) |
| *Sambuccus comp. (Sambucus nigra e medulla D3, Sambucus nigra ex umbella D2, Terebinthina D4)* | *PO* | 1 (0.41%) |
| *Stibium metallicum praeparatum D6* | *PO* | 1 (0.41%) |
|  | *IV* | 3 (1.22%) |
| *Tartarus stibiatus (potency not specified)* | *PO* | 28 (11.38%) |
|  | *IV* | 20 (8.13%) |
|  | *IV + PO* | 8 (3.25%) |
|  | *IV + Inhalation* | 1 (0.41%) |
|  | *Not Mentioned* | 2 (0.81%) |
| *Urtica comp. (Conchae D6, Stannum D9, Urtica urens D2)* | *PO* | 1 (0.41%) |
